# Specific IgE response to different grass pollen allergen components in children undergoing sublingual immunotherapy

**DOI:** 10.1186/1476-7961-10-7

**Published:** 2012-06-13

**Authors:** Francesco Marcucci, Laura Sensi, Cristoforo Incorvaia, Ilaria Dell’Albani, Giuseppe Di Cara, Franco Frati

**Affiliations:** 1 Spin-Off ATRP Srl, Allergic Tests Research and Production, Perugia, Italy; 2 Allergy/Pulmonary rehabilitation, ICP Hospital, Milan, Italy; 3 Medical and Scientific Department, Stallergenes, Milan, Italy; 4 Institute of Pediatrics, Department of Medical and Surgical specialty and Public Health, Perugia, Italy

## Abstract

**Background:**

Grass pollen is a major cause of respiratory allergy worldwide and contain a number of allergens, some of theme (Phl p 1, Phl p 2, Phl p 5, and Phl 6 from *Phleum pratense*, and their homologous in other grasses) are known as major allergens. The administration of grass pollen extracts by immunotherapy generally induces an initial rise in specific immunoglobulin E (sIgE) production followed by a progressive decline during the treatment. Some studies reported that immunotherapy is able to induce a *de novo* sensitisation to allergen component previously unrecognized.

**Methods:**

We investigated in 30 children (19 males and 11 females, mean age 11.3 years), 19 treated with sublingual immunotherapy (SLIT) by a 5-grass extract and 11 untreated, the sIgE and sIgG4 response to the different allergen components.

**Results:**

Significant increases (p < 0.001) were detected for Phl p 1, Phl p 2, Phl p 5, and Phl p 6, while sIgE levels induced in response to Phl p 7 and Phl p 12 were low or absent at baseline and unchanged following SLIT treatment; no new sensitisation was detected. As to IgG4, significant increases were found for Phl p2 and Phl p 5, while the increase for Phl p 12 was not significant. In the control group, no significant increase in sIgE for any single allergen component was found.

**Conclusions:**

These findings confirm that the initial phase of SLIT with a grass pollen extract enhances the sIgE synthesis and show that the sIgE response concerns the same allergen components which induce IgE reactivity during natural exposure.

## Introduction

Grass pollen is a major cause of respiratory allergy worldwide [1,2]. Grasses are botanically classified in the family of *Gramineae* and in particular in the subfamily of *Pooideae*, which includes the species most commonly responsible of allergic sensitisation, such as *Anthoxantum odoratum*, *Dactylis glomerata*, *Festuca elatior*, *Holcus lanatus*, *Lolium perenne*, *Phleum pratense*, and *Poa pratensis*. Another species causing respiratory allergy is *Cynodon dactylon,* which belongs to the subfamily of *Panicoideae*. These species have variable importance in different geographical areas. In Europe, *Dactylis glomerata*, *Poa pratensis*, *Lolium perenne* and *Anthoxantum odoratum* are homogeneously distributed, while *Phleum pratense* is the dominant grass in Northern regions and in the United Kingdom, and *Cynodon dactylon* is present only in Southern regions [1]. To date, more than 150 allergens from 52 grass species are known, classified in 13 groups according to similar physicochemical and immunologic properties [3,4]. Among these allergens, the molecules from *Phleum pratense* Phl p 1, Phl p 2, Phl p 5, and Phl 6, and their homologous in other grasses, are known as major allergens [4]. The administration of a pollen allergen extract by specific immunotherapy with conventional schedules generally induces an initial rise in specific immunoglobulin E (sIgE) production followed by a progressive decline during the treatment. This event was first observed in 1971 [5] and confirmed in a number of studies using subcutaneous immunotherapy (SCIT), being reasonable to presume that the early rise occurs because sIgE synthesis is stimulated when tolerogenic mechanisms of immunotherapy (such as the generation of IgG-blocking antibodies and changes in T cell subpopulations) are not yet developed; subsequently, when these mechanisms are initiated, sIgE synthesis is inhibited [6]. Recent studies on sublingual immunotherapy (SLIT) with administration of adequate allergen doses have shown a pattern of sIgE response analogous to that observed for SCIT [7-9], and this agrees with the fact that the two treatments share similar mechanisms of action [6,10]. Only a few studies evaluated the sIgE response to single allergen components during immunotherapy with grass pollen extract [11-14] and only one investigated this issue in SLIT with a *Phleum pratense* extract in adults [14]. We evaluated the sIgE and sIgG4 response to the different allergen components in children treated with SLIT using a 5-grass pollen extract.

## Materials and methods

### Patients

The study included 30 children (19 males and 11 female, mean age 11.3 years) with grass pollen allergy, presenting as persistent allergic rhinitis for at least 2 consecutive years and graded as moderate/severe according to the Allergic Rhinitis and its Impact on Asthma (ARIA) classification [15]. Of them, 19 underwent SLIT with a 5-grass pollen extract standardized in Index of Reactivity (IR) (Staloral 300 IR, Stallergenes, Antony, France), while 11 patients were not treated and served as controls. SLIT was performed by the build-up schedule in four days (30 – 60 – 120 – 240 IR) suggested by the manufacturer starting in the month of February. A maintenance dose of 240 IR was then administered 3 times a-week until the end of June. All side effects were recorded in diary cards. The study was approved by the Ethics Committee of the University of Perugia.

### In vitro methods

The IgE reactivity to the single grass pollen allergen components (Phl p 1, Phl p 2, Phl p 5, Phl p 6, Phl p 7, and Phl p 12) was determined using ImmunoCAP® (Phadia AB, Uppsala, Sweden) tests; IgG4 reactivity to Phl p 2, Phl p 5 and Phl p 12 was also measured. sIgE and sIgG4 measurement was performed before starting SLIT and at the end of the pollen season.

### Statistical analysis

Wilcoxon signed rank test for paired data was used to detect differences between baseline and after SLIT values of specific IgE and IgG antibodies for recombinant epitopes of *Phleum pratense*. A p value lower than 0.05 was stated as significant.

## Results

**Figure 1:**
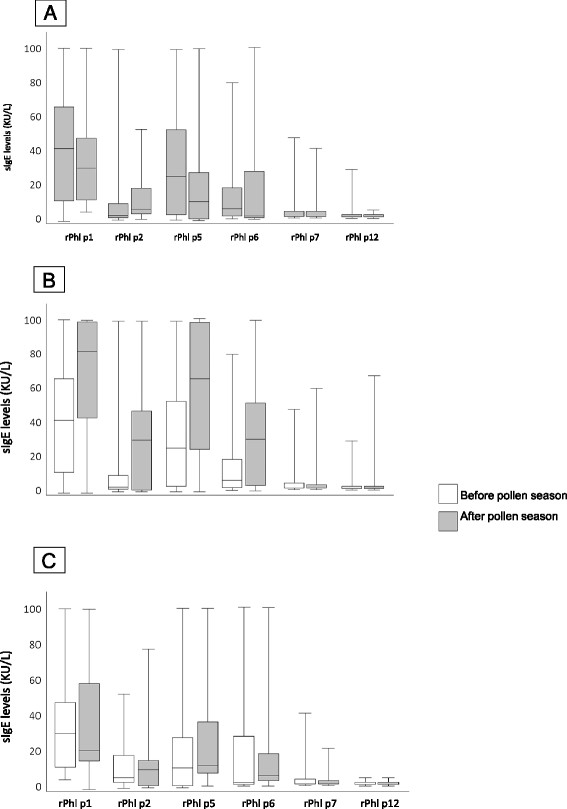
** Basal values of sIgE in pre-SLIT and control group (A) and changes in sIgE towards *Phleum *epitopes, before and after pollen season in patients treated by grass SLIT (B) and in untreated control patients (C).**

**Figure 2:**
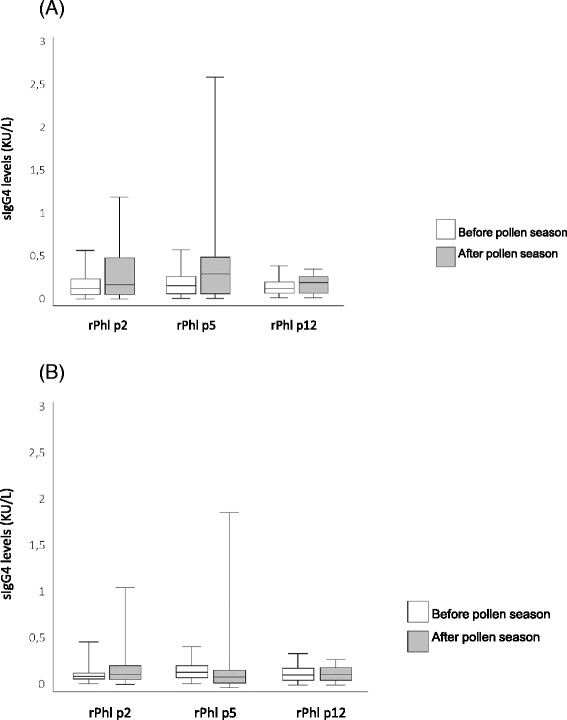
sIgG4 sensitization in patients treated by grass SLIT (A) and in untreated control patients (B).

The SLIT treatment was well tolerated, only local reactions in the mouth were observed in 9 of the 19 treated patients (47%), no systemic reaction occurred. Figure 1 shows the basal values of sIgE in both groups and the mean changes in sIgE to grass allergen components in SLIT treated children and in controls. Basal values were not significantly different between SLIT treated and control patients. Significant increases (p < 0.001) were detected for Phl p 1, Phl p 2, Phl p 5, and Phl p 6, while sIgE levels induced in response to Phl p 7 and Phl p 12 were low or absent at baseline and unchanged following SLIT treatment. In the control group, no significant increase in sIgE for any single allergen component was found. A significant increase of sIgG4 was detected for Phl p 2 (p = 0.009), and Phl p 5 (p = 0.025), while sIgG4 for Phl p 12 showed a not significant increase (Figure 2). Most patients had a similar increase in sIgE and sIgG4 reactivity, only one patient showed a slight increase of sIgG4 to Phl p 12 (from 0.14 to 0.21 mg/L) with no change in sIgE levels. No new sIgE sensitizations were found in SLIT group.

## Discussion

Of the 13 allergen groups identified in grass pollen, four – Phl p 4, 7, 11, and 12 – are not grass-specific, while the other groups are grass-specific. Considering the classification of allergens as major or minor depending on their recognition by IgE antibodies from more or less than 50% of sensitised patients, respectively, Group 1 and Group 5 contain the major grass pollen allergens. Group 1 allergens are acidic glycoproteins, with a m.w. of 31–35 kDA, and have high homology (about 90% of the amino acid sequence) [16], while Group 5 allergens are proteins showing ribonuclease activity, occurring in two non-glycosilated isoforms, with a m.w. of 27–33 kDa, which have a lesser homology due to a 25-30% divergence in amino acid sequence [4]. Instead, Phl p 7, a calcium-binding protein, and Phl p 12, a profilin, are ubiquitous molecules showing cross-reactivity in a number of allergen sources that are not naturally found as specific grass pollen allergens [4]. Due to the complex allergen repertoire of grass pollen, the pattern of sIgE response of sensitised individual is largely variable, especially concerning the recognition of the different epitopes expressed in the various allergens. The sensitisation is obviously influenced by the kind of exposure and particularly by the distribution of the different grasses in the geographical area where the patients live. For example, *Phleum pratense* clearly prevails in Northern Europe, while is less present in central and southern Italy, as demonstrated by phenologic studies [17]. The contact of the immunologic system with the allergens introduced by means of specific immunotherapy is a different kind of stimulation, that is potentially able to induce a sIgE response to allergen components previously not recognized by natural exposure. This was true in the study by Ball et al, who found a *de novo* induction of sIgE against new allergens in one of 8 patients treated with SCIT with a grass pollen extract. This finding lead the authors to suggest the *de novo* sensitisation as a factor able to explain the unpredictability of specific immunotherapy performed with allergen extracts [11]. Similar observations were reported by Moverare et al. in a larger group of 34 patients allergic to birch pollen treated with SCIT, 29% of whom developed new sensitizations to rBet v 2 and/or rBet v 4, though the sIgE levels were low and the clinical relevance was not known [18]. Indeed, other investigations on grass-allergic patients, namely 33 subjects from North-West Italy [12] and 19 subjects from Germany [13] treated with SCIT did not find any new sensitisation. The only available study on sIgE profiles to grass pollen allergens during SLIT was performed on 40 adults treated with a *Phleum pratense* extract in tablets. Most patients had low titers of sIgE to Phl p 1 and Phl p 5 before SLIT and showed a dose-dependent increase during the treatment, while sIgE titers to Phl p 7 and Phl p 12 were very low both before and after SLIT; no new sensitisation was detected [14].

We addressed the present study to evaluate the changes in sIgE levels to Phl p 1, Phl p 2, Phl p 5, Phl p 6, Phl p 7, and Phl p 12 in children treated with SLIT using a 5-grass extract which was demonstrated to be immunologically effective [19,20]. Significant increases were detected for Phl p 1, Phl p 2, Phl p 5, and Phl p 6, but not for Phl p 7 and Phl p 12, which maintained the pre-treatment low levels. We confirmed the lack of new sensitisations, and this is important because the immunologic stimulation given by a 5-grass extract is wider than that by an extract with a single grass. Also sIgG4 were measured, with detection of significant increase for Phl p 2 and Phl p 5 but not for Phl p 12. Concerning the latter, the mild increase (from 0.14 to 0.21 mg/ml) excludes that the low level of sIgE was influenced by the blocking activity of IgG4. For the other allergens, a substantially parallel pattern of IgE and IgG4 response was found. This result is in agreement with previous observations [14]. Our results cannot be generalized, considering the extreme heterogeneity of the immune-response to grass pollen, as recently reported by Tripodi et al. [21]. In particular, it is not known whether the production of sIgE to the different allergen components is influenced by only genetic predisposition or also by the kind of allergen exposure. In addition, it remains to be investigated if a prolonged period of observation during SLIT could detect changes in sIgE specificities not occurring in early phases of treatment.

## Conclusions

The findings of this study confirm that the initial phase of SLIT with a grass pollen extract enhances the sIgE synthesis and show that the sIgE response concerns the same allergen components which induce IgE reactivity during natural exposure. The observation seems important where IgE response to individual pollen components is concerned and warrants further research to confirm and expand the knowledge on this issue.

## Competing interest

Francesco Marcucci and Cristoforo Incorvaia are scientific consultants for Stallergenes Italy.

Ilaria Dell’Albani and Franco Frati are employees of Stallergenes Italy.

## Authors’ contribution

FM conceived the study, participated in its design and analysed the results. LS participated in the study design and carried out the tests. CI analysed the results and participated to writing the manuscript. ID participated to writing the manuscript. GD carried out the tests, analysed the results and participated to writing the manuscript. FF participated in the study design, analysed the results and participated to writing the manuscript. All authors read and approved the final manuscript.
